# Maintenance treatment with rucaparib for recurrent ovarian carcinoma in ARIEL3, a randomized phase 3 trial: The effects of best response to last platinum‐based regimen and disease at baseline on efficacy and safety

**DOI:** 10.1002/cam4.4260

**Published:** 2021-09-21

**Authors:** Ana Oaknin, Amit M. Oza, Domenica Lorusso, Carol Aghajanian, Andrew Dean, Nicoletta Colombo, Johanne I. Weberpals, Andrew R. Clamp, Giovanni Scambia, Alexandra Leary, Robert W. Holloway, Margarita Amenedo Gancedo, Peter C. Fong, Jeffrey C. Goh, David M. O’Malley, Deborah K. Armstrong, Susana Banerjee, Jesus García‐Donas, Elizabeth M. Swisher, Terri Cameron, Lara Maloney, Sandra Goble, Jonathan A. Ledermann, Robert L. Coleman

**Affiliations:** ^1^ Gynaecologic Cancer Programme Vall d’Hebron Institute of Oncology (VHIO), Hospital Universitari Vall d’Hebron, Vall d’Hebron Barcelona Hospital Campus Barcelona Spain; ^2^ Division of Medical Oncology and Hematology Princess Margaret Cancer Centre University Health Network Toronto Canada; ^3^ Multicentre Italian Trials in Ovarian Cancer and Gynecologic Malignancies and Gynecologic Oncology Unit Fondazione IRCCS, Istituto Nazionale dei Tumori Milan Italy; ^4^ Department of Medicine Memorial Sloan Kettering Cancer Center New York New York USA; ^5^ Oncology St John of God Subiaco Hospital Subiaco Western Australia Australia; ^6^ Gynecologic Cancer Program University of Milan‐Bicocca and European Institute of Oncology IRCCS Milan Italy; ^7^ Division of Gynecologic Oncology Ottawa Hospital Research Institute Ottawa Canada; ^8^ Department of Medical Oncology The Christie NHS Foundation Trust and University of Manchester Manchester United Kingdom; ^9^ Gynecologic Oncology Unit Fondazione Policlinico Universitario A. Gemelli IRCCS and Scientific Directorate Rome Italy; ^10^ Gynecological Unit, Gustave Roussy Cancer Center INSERM U981, and Groupe d’Investigateurs Nationaux pour l’Etude des Cancers Ovariens Villejuif France; ^11^ Gynecologic Oncology AdventHealth Cancer Institute Orlando Florida USA; ^12^ Medical Oncology Department Oncology Center of Galicia, Doctor Camilo Veiras La Coruña Spain; ^13^ Medical Oncology Department Auckland City Hospital, and University of Auckland Auckland New Zealand; ^14^ Department of Oncology, Cancer Care Services Royal Brisbane and Women’s Hospital, and University of Queensland Herston Queensland Australia; ^15^ Division of Gynecologic Oncology The Ohio State University, James Cancer Center Columbus Ohio USA; ^16^ Oncology, Gynecology and Obstetrics Johns Hopkins Kimmel Cancer Center Baltimore Maryland USA; ^17^ Gynaecology Unit The Royal Marsden NHS Foundation Trust and Institute of Cancer Research London United Kingdom; ^18^ Division of Medical Oncology HM Hospitales—Centro Integral Oncológico Hospital de Madrid Clara Campal Madrid Spain; ^19^ Division of Gynecologic Oncology University of Washington Seattle Washington USA; ^20^ Clinical Science Clovis Oncology UK Ltd Cambridge United Kingdom; ^21^ Clinical Development Clovis Oncology, Inc Boulder Colorado USA; ^22^ Biostatistics Clovis Oncology, Inc Boulder Colorado USA; ^23^ Department of Oncology University College London (UCL) Cancer Institute and UCL Hospitals London United Kingdom; ^24^ Department of Gynecologic Oncology and Reproductive Medicine University of Texas MD Anderson Cancer Center Houston Texas USA; ^25^ Present address: Gynecologic Oncology Unit Fondazione Policlinico Universitario A. Gemelli IRCCS and Scientific Directorate Rome Italy; ^26^ Present address: US Oncology Research The Woodlands Texas USA

**Keywords:** clinical trials, gynecological oncology, medical oncology, target therapy, women's cancer

## Abstract

**Background:**

The efficacy and safety of rucaparib maintenance treatment in ARIEL3 were evaluated in subgroups based on best response to most recent platinum‐based chemotherapy and baseline disease.

**Methods:**

Patients were randomized 2:1 to receive either oral rucaparib at a dosage of 600 mg twice daily or placebo. Investigator‐assessed PFS was assessed in prespecified, nested cohorts: *BRCA*‐mutated, homologous recombination deficient (HRD; *BRCA* mutated or wild‐type *BRCA*/high loss of heterozygosity), and the intent‐to‐treat (ITT) population.

**Results:**

Median PFS for patients in the ITT population with a complete response to most recent platinum‐based chemotherapy was 11.1 months in the rucaparib arm (126 patients) versus 5.6 months in the placebo arm (64 patients) (HR, 0.33 [95% CI, 0.23–0.48]), and in patients with a partial response (249 vs. 125), it was 9.0 versus 5.3 months (HR, 0.38 [0.30–0.49]). In subgroups of the ITT population based on baseline disease, median PFS was 8.2 versus 5.3 months (HR, 0.40 [0.28–0.57]) in patients with measurable disease (141 rucaparib vs. 66 placebo), 10.4 versus 4.5 months (HR, 0.31 [0.20–0.48]) in those with nonmeasurable but evaluable disease (104 vs. 56), and 14.1 versus 7.3 months (HR, 0.35 [0.24–0.51]) in those with no residual disease (130 vs. 67). Across subgroups, significantly longer median PFS was observed with rucaparib versus placebo in the *BRCA*‐mutated and HRD cohorts. Objective responses were reported in patients with measurable disease and in patients with nonmeasurable but evaluable baseline disease. Safety was consistent across subgroups.

**Conclusion:**

Rucaparib maintenance treatment provided clinically meaningful efficacy benefits across subgroups based on response to last platinum‐based chemotherapy or baseline disease.

## Graphical abstract

The efficacy and safety of the PARP inhibitor rucaparib as maintenance treatment for recurrent ovarian cancer were similar regardless of whether patients had a complete or partial response to their last platinum‐based chemotherapy or according to whether they had measurable, nonmeasurable but evaluable, or no residual disease at baseline. Rucaparib also reduced the disease burden in patients who had measurable or nonmeasurable but evaluable disease at baseline.

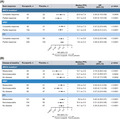

## 1 INTRODUCTION

For patients with advanced ovarian cancer, initial treatment has historically consisted of surgery followed by chemotherapy (platinum and/or taxane); upon completion of chemotherapy, patients would be followed closely until relapse (active surveillance).^1^, ^2^ Most patients experience disease recurrence and would typically receive further chemotherapy followed by active surveillance, with efficacy rapidly declining in successive lines of treatment.^3^

Recently, maintenance with targeted therapies following a complete response (CR) or partial response (PR) to chemotherapy has become a standard‐of‐care option for patients with advanced ovarian cancer.^1^, ^2^ Maintenance treatment aims to extend progression‐free survival (PFS) following a response to chemotherapy by delaying disease progression. Maintenance treatment also aims to delay the need for subsequent chemotherapy to reduce toxicities associated with these therapies; such toxicities may have a negative impact on the quality of life for patients.^4^, ^5^, ^6^ In Europe and the United States, bevacizumab (angiogenesis inhibitor), olaparib (poly[ADP‐ribose] polymerase [PARP] inhibitor), niraparib (PARP inhibitor), and the combination of bevacizumab and olaparib are approved for first‐line maintenance treatment.^7^ Maintenance treatment is also important in the recurrent setting; bevacizumab, olaparib, niraparib, and rucaparib (PARP inhibitor) are each approved as maintenance treatment in this setting in both Europe and the United States.^7^

Although current guidelines recommend maintenance treatment for eligible patients whose ovarian cancer has responded to prior chemotherapy,^1^, ^2^ it remains unclear how clinical factors, such as depth of response to prior therapy or level of residual tumor burden, should play a role in the decision‐making process.^8^ For example, prior studies have shown that the presence of residual disease reduces the clinical benefit of second‐line chemotherapy in patients with recurrent ovarian cancer,^9^ but data evaluating the association of residual disease or similar factors with the magnitude of clinical benefit that patients with recurrent ovarian cancer derive from maintenance treatment are currently limited.^10^, ^11^, ^12^, ^13^ Therefore, we sought to extend our understanding utilizing data from the ARIEL3 study (NCT01968213).

In ARIEL3, the pivotal study of rucaparib maintenance treatment in patients with recurrent ovarian cancer, there was a significant improvement in investigator‐assessed PFS (primary endpoint) and blinded independent central review (BICR)‐assessed PFS (secondary endpoint) with rucaparib versus placebo. Improvements were seen all in three prespecified, nested cohorts, including in the overall intent‐to‐treat (ITT) population.^14^

Here, we present analyses of data from ARIEL3 evaluating two key subgroups based on best response to most recent platinum‐based chemotherapy and disease status at baseline, according to Response Evaluation Criteria in Solid Tumors (RECIST) v1.1. Limited data regarding PFS in the ITT population for these subgroups were published previously.^14^ We now further expand on that analysis to include an examination of PFS for each subgroup within the nested *BRCA*‐mutant and homologous recombination deficient (HRD; *BRCA* mutated or wild‐type *BRCA*/high loss of heterozygosity) cohorts, which represent patient populations of particular interest in gynecologic oncology. Additionally, for the first time, we present analysis of safety outcomes for these two subgroups. Notably, the previous publication provided a subgroup analysis of PFS based on the binary presence or absence of measurable disease. However, as understanding the impact of any evaluable residual disease on patient outcomes is of interest, we have re‐evaluated disease status at baseline to include three distinct subgroups (measurable residual disease vs. nonmeasurable but evaluable residual disease vs. no residual disease). This refined analysis facilitated examination of PFS and objective response in patients with nonmeasurable but evaluable disease. These exploratory subgroup analyses evaluate the benefits of maintenance treatment with rucaparib versus placebo (which is equivalent to active surveillance) in patients with a CR and/or no disease and in patients with a PR and/or measurable/nonmeasurable but evaluable disease, including the evaluation of clinical benefits, such as longer PFS and deepening of response.

## 2 METHODS

### 2.1 Study design and patients

The randomized, double‐blind, phase 3 ARIEL3 trial was conducted in 11 countries at 87 hospitals and cancer centers. Patients were enrolled between 7 April 2014 and 19 July 2016. The study was approved by national or local institutional review boards and performed in accordance with the Declaration of Helsinki and Good Clinical Practice Guidelines of the International Council for Harmonisation. Patients provided written informed consent before participating in the study. A CONSORT diagram and full study design details, including all inclusion/exclusion criteria, screening and randomization procedures, and a description of drug administration and dosing were reported in the primary publication^14^; key points of the study design and procedures are provided in the supporting information.

### 2.2 Outcomes

Investigator‐assessed PFS, the primary outcome of ARIEL3, was defined as the time from randomization to investigator‐assessed disease progression per RECIST v1.1 or death and has been previously reported.^14^ Investigator‐assessed PFS and BICR‐assessed PFS were analyzed in patient subgroups using the primary efficacy data after unblinding, at which time the PFS data were mature (visit cutoff 15 April 2017). Subgroup analyses were conducted by best response to prior chemotherapy (CR vs. PR) and disease at baseline (measurable disease vs. nonmeasurable but evaluable disease vs. no disease). The measurable disease subgroup included patients with measurable target lesions (with or without nontarget lesions) per RECIST v1.1 at baseline (investigator assessed). The nonmeasurable but evaluable disease subgroup included patients without target lesions identified but with evidence of nontarget disease per RECIST v1.1 (e.g., lesions <10 mm, ascites, pleural or pericardial effusion) at baseline (investigator assessed).

Safety was evaluated using a visit cutoff date of 31 December 2019. Relative risk for any grade and grade ≥3 treatment‐emergent adverse events (TEAEs) of key interest (alanine aminotransferase [ALT] elevation and/or aspartate aminotransferase [AST] elevation [combined preferred terms], anemia and/or decreased hemoglobin [combined preferred terms], asthenia and/or fatigue [combined preferred terms], nausea, thrombocytopenia and/or decreased platelet count [combined preferred terms], and vomiting) were determined in each subgroup category.

Data for prespecified, investigator‐assessed exploratory postprogression endpoints (visit cutoff 31 December 2017) in the three prespecified, nested cohorts have been reported elsewhere.^15^ The prespecified number of events needed for reporting overall survival (secondary endpoint) has not been reached; this endpoint will be reported once the data are mature.

### 2.3 Statistical analyses

The rationale for target enrollment in ARIEL3 was described previously.^14^ For each subgroup category, analyses were conducted in the three prespecified, nested cohorts. PFS was evaluated using Kaplan‐Meier methodology; additional details on statistical methodology are provided in the supporting information. Treatment‐by‐subgroup interaction tests were performed using a Cox proportional hazards model. *p* values for these exploratory subgroup analyses are presented for descriptive purposes only.

An exploratory analysis evaluated investigator‐assessed objective response (per RECIST v1.1) in patients with measurable disease at baseline (response defined as achieving a CR or PR) or nonmeasurable disease at baseline (response defined as achieving a CR). SAS version 9.4 (SAS Institute, Cary, NC, USA) was used to perform statistical analyses.

## 3 RESULTS

### 3.1 Patients

Five hundred and sixty‐four patients were enrolled and randomized to rucaparib (*n *= 375) or placebo (*n* = 189). The majority of patients had a PR (rucaparib, *n *= 249; placebo, *n* = 125) versus a CR (*n* = 126; *n* = 64) to prior platinum‐based chemotherapy (Table S1A). A similar number of patients had measurable disease (rucaparib, *n* = 141; placebo, *n* = 66), nonmeasurable disease (*n* = 104; *n *= 56), and no disease at baseline (*n* = 130; *n* = 67; Table S1B). In each subgroup, baseline demographics and characteristics, as well as prior treatment history were generally well balanced across the rucaparib and placebo arms.

### 3.2 Efficacy

**FIGURE 1:**
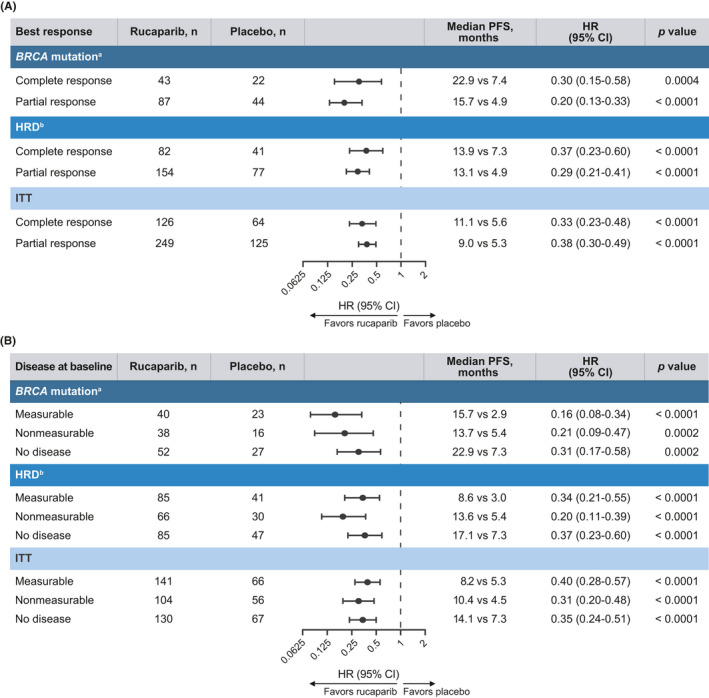
Investigator‐assessed PFS analyses in subgroups. Subgroups defined by (A) best response to last platinum‐based chemotherapy regimen and (B) disease at baseline. *p* values are presented for descriptive purposes only. ^a^Germline, somatic or unknown; ^b^ *BRCA* mutation + wild‐type *BRCA*/high loss of heterozygosity. CI indicates confidence interval; HR, hazard ratio; HRD, homologous recombination deficient; ITT, intent to treat; PFS, progression‐free survival

**FIGURE 2:**
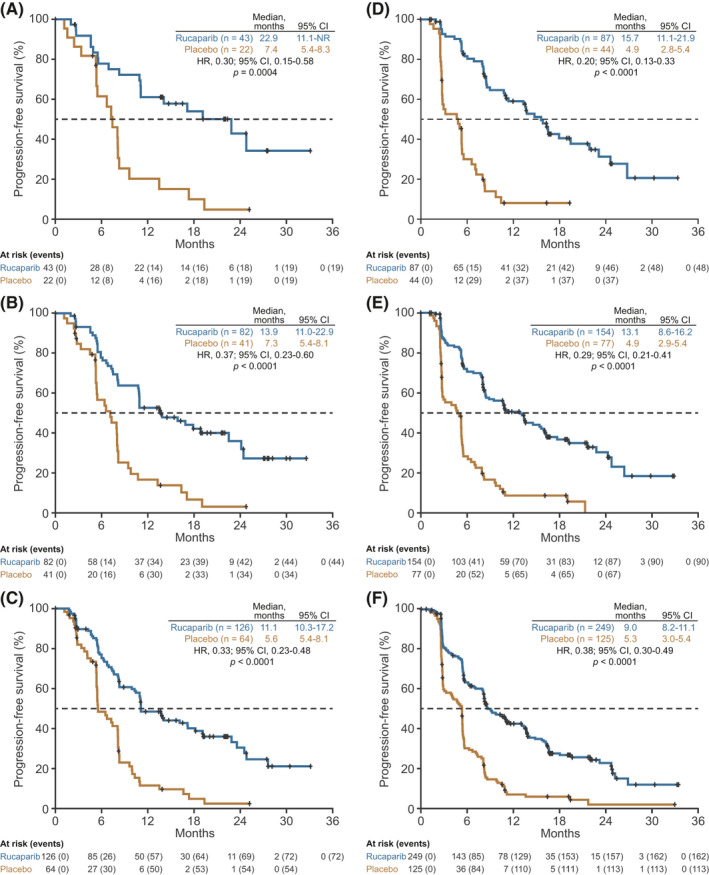
Investigator‐assessed PFS according to best response to last platinum‐based chemotherapy. Patients with a CR to last platinum‐based chemotherapy in the (A) *BRCA*‐mutated cohort, (B) HRD cohort, and (C) ITT population. Patients with a PR to last platinum‐based chemotherapy in the (D) *BRCA*‐mutated cohort, (E) HRD cohort, and (F) ITT population. *p* values were nonsignificant for treatment by best response subgroup (CR vs. PR) interaction tests (*BRCA*‐mutated cohort, *p* = 0.5680; HRD cohort, *p* = 0.4029; ITT population, *p* = 0.7001). *P* values are presented for descriptive purposes only. CI indicates confidence interval; CR, complete response; HR, hazard ratio; HRD, homologous recombination deficient; ITT, intent to treat; NR, not reached; PFS, progression‐free survival; PR, partial response

Across all prespecified analysis cohorts, irrespective of best response to most recent platinum‐based therapy, the median duration of investigator‐assessed PFS was significantly longer in the rucaparib arm versus the placebo arm (Figures 1A and 2A–F). In the ITT population, median duration of PFS was 11.1 months in the rucaparib arm compared with 5.6 months in the placebo arm (2.0‐fold longer) among patients with a CR (hazard ratio [HR], 0.33; 95% confidence interval [CI], 0.23–0.48; *p* < 0.0001; Figures 1A and 2C), and it was 9.0 months compared with 5.3 months (1.7‐fold longer), respectively, among patients with a PR (HR, 0.38; 95% CI, 0.30–0.49; *p* < 0.0001; Figures 1A and 2F). Significant extensions in PFS with rucaparib versus placebo were also observed in the *BRCA*‐mutated cohort (3.1‐ and 3.2‐fold longer median PFS in the CR and PR subgroups; Figures 1A, 2A, and 2D) and the HRD cohort (1.9‐ and 2.7‐fold longer, respectively; Figures 1A, 2B, and 2E). Similar findings were observed for BICR‐assessed PFS in all nested cohorts (Figure S1A–F). No significant treatment‐by‐best response to most recent platinum‐based chemotherapy subgroup (CR vs. PR) interactions was observed for investigator‐ or BICR‐assessed PFS in any of the nested cohorts.

**FIGURE 3:**
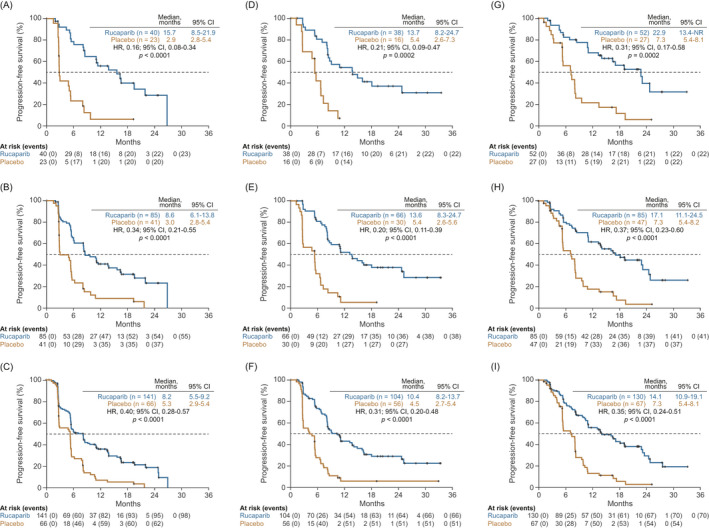
Investigator‐assessed PFS according to disease at baseline. Patients with measurable disease at baseline in the (A) *BRCA*‐mutated cohort, (B) HRD cohort, and (C) ITT population. Patients with nonmeasurable but evaluable disease at baseline in the (D) *BRCA*‐mutated cohort, (E) HRD cohort, and (F) ITT population. Patients with no disease at baseline in the (G) *BRCA*‐mutated cohort, (H) HRD cohort, and (I) ITT population. *p* values were nonsignificant for treatment by baseline disease subgroup interaction tests (*BRCA*‐mutated cohort: no disease vs. nonmeasurable disease, *p* = 0.3153; no disease vs. measurable disease, *p* = 0.2078; nonmeasurable disease vs. measurable disease, *p* = 0.6793; HRD cohort: no disease vs. nonmeasurable disease, *p* = 0.7447; no disease vs. measurable disease, *p* = 0.8119; nonmeasurable disease vs. measurable disease, *p* = 0.1317; ITT population: no disease vs. nonmeasurable disease, *p* = 0.4510; no disease vs. measurable disease, *p* = 0.1920; nonmeasurable disease vs. measurable disease, *p* = 0.3953). *p* values are presented for descriptive purposes only. CI indicates confidence interval; HR, hazard ratio; HRD, homologous recombination deficient; ITT, intent to treat; NR, not reached; PFS, progression‐free survival

In subgroups defined according to baseline disease status, median PFS as assessed by the investigator was significantly longer with rucaparib than with placebo irrespective of whether patients had measurable, nonmeasurable but evaluable, or no disease across all three prespecified analysis cohorts (Figures 1B and 3A–I). Among patients with measurable disease in the ITT population, median investigator‐assessed PFS was 1.5‐fold longer in the rucaparib arm compared with the placebo arm (8.2 vs. 5.3 months; HR, 0.40; 95% CI, 0.28–0.57; *p* < 0.0001; Figures 1B and 3C). In those with nonmeasurable but evaluable disease, investigator‐assessed median PFS was 2.3‐fold longer (10.4 vs. 4.5 months; HR, 0.31; 95% CI, 0.20–0.48; *p* < 0.0001; Figures 1B and 3F), and in patients with no disease, investigator‐assessed median PFS was 1.9‐fold longer (14.1 vs. 7.3 months; HR, 0.35; 95% CI, 0.24–0.51; *p* < 0.0001; Figures 1B and 3I). In the *BRCA* cohort, median PFS was significantly longer in the rucaparib arm than the placebo arm (5.4‐, 2.5‐, and 3.1‐fold longer in patients with measurable, nonmeasurable, and no disease, respectively; Figures 1, 3, 1, 3, 1, 3, and 1, 3); similar results were also seen in the HRD cohort (2.9‐, 2.5‐, and 2.3‐fold longer, respectively; Figures 1, 3, 1, 3, 1, 3, and 1, 3). Median BICR‐assessed PFS was also significantly longer in the rucaparib arm compared with the placebo arm in all nested cohorts (Figures S2A–I). No significant treatment‐by‐baseline disease subgroup interactions were observed for investigator‐ or BICR‐assessed PFS in any of the nested cohorts.

Confirmed RECIST v1.1 responses were reported in patients with measurable as well as in patients with nonmeasurable but evaluable disease at baseline (Table 1). In the ITT population, a CR or PR was observed in 26/141 (18.4%) patients receiving rucaparib and 5/66 (7.6%) patients receiving placebo who had measurable disease. Among patients in the ITT population with nonmeasurable disease, 23/104 (22.1%) patients receiving rucaparib achieved a confirmed CR during the study (which includes seven patients without a *BRCA* mutation or high loss of heterozygosity); 2/56 (3.6%) patients in this subgroup who received placebo achieved a confirmed CR.

**TABLE 1:** Confirmed ORR in patients with investigator‐assessed measurable or nonmeasurable but evaluable disease at baseline

Confirmed ORR, *n*/*N* (%) [95% CI]	*BRCA* mutated		HRD		ITT	
	Rucaparib	Placebo	Rucaparib	Placebo	Rucaparib	Placebo
Measurable disease at baseline^14^	15/40 (37.5) [22.7–54.2]	2/23 (8.7) [1.1–28.0]	23/85 (27.1) [18.0–37.8]	3/41 (7.3) [1.5–19.9]	26/141 (18.4) [12.4–25.8]	5/66 (7.6) [2.5–16.8]
Nonmeasurable disease at baseline	12/38 (31.6) [17.5–48.7]	1/16 (6.3) [0.2–30.2]	16/66 (24.2) [14.5–36.4]	2/30 (6.7) [0.8–22.1]	23/104 (22.1) [14.6–31.3]	2/56 (3.6) [0.4–12.3]

Abbreviations: CI, confidence interval; HRD, homologous recombination deficient; ITT, intent to treat; ORR, objective response rate.

### 3.3 Safety

In both the rucaparib and placebo arms, almost all patients reported at least one any‐grade TEAE (Table S2). Among rucaparib‐treated patients, the most frequent any‐grade TEAEs were nausea, asthenia and/or fatigue, anemia and/or decreased hemoglobin, constipation, dysgeusia, and vomiting. Anemia and/or decreased hemoglobin was the most frequent grade ≥3 TEAE. TEAEs of myelodysplastic syndrome or acute myeloid leukemia were reported in five (1.3%) rucaparib‐treated patients; no cases were reported in the placebo arm.

**FIGURE 4:**
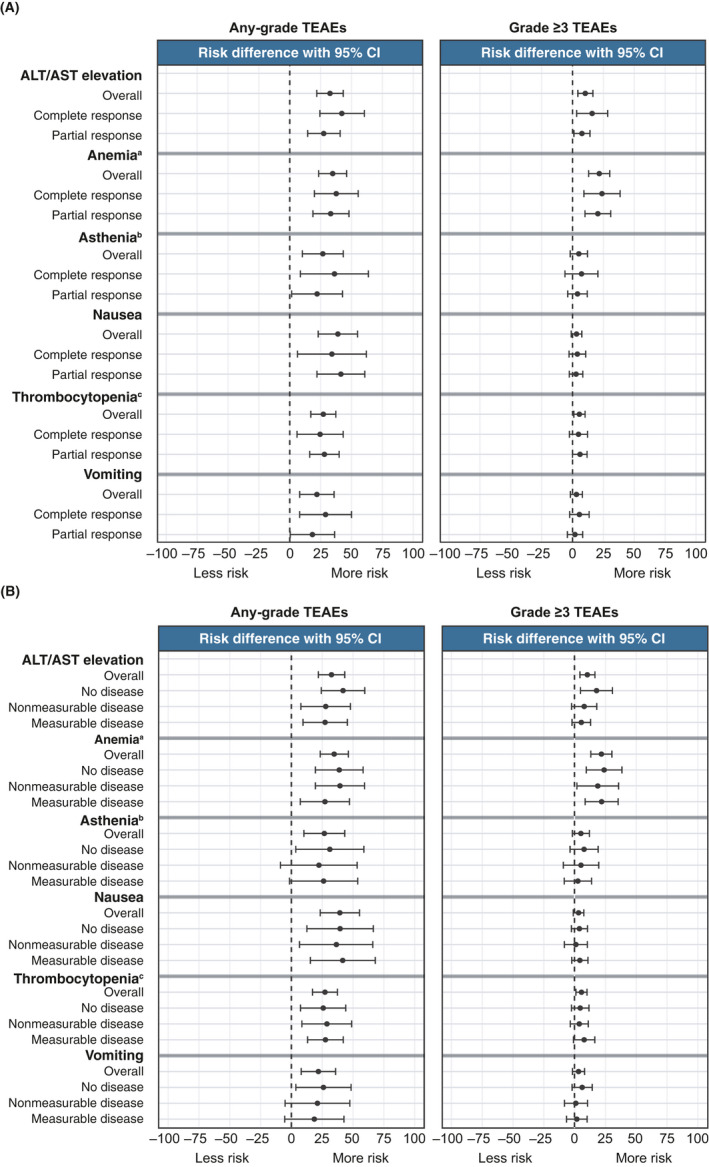
Relative risk of all‐grade and grade ≥3 TEAEs of key interest in subgroups. Subgroups defined by (A) best response to last platinum‐based chemotherapy and (B) disease at baseline. ^a^Anemia and/or decreased hemoglobin; ^b^Asthenia and/or fatigue; ^c^Thrombocytopenia and/or platelet count decreased. ALT indicates alanine aminotransferase; AST, aspartate aminotransferase; CI, confidence interval; TEAE, treatment‐emergent adverse event

For patients with a CR or PR, the relative risks of any‐grade TEAEs of key interest were higher with rucaparib versus placebo, with no differences in risk evident between the two subgroups (Figure 4A). For grade ≥3 TEAEs of key interest, relative risks were also similar for patients with a CR or PR. Grade ≥3 ALT and/or AST elevation and anemia and/or decreased hemoglobin had higher relative risk with rucaparib versus placebo. Among patients who received rucaparib, the incidence of treatment interruptions and/or dose reductions (i.e., dose modifications) and treatment discontinuations was similar between the CR and PR subgroups (Table S2A). Excluding progression, 1/125 (0.8%) rucaparib‐treated patients with a CR at baseline, 5/247 (2.0%) rucaparib‐treated patients with a PR at baseline, and 1/64 (1.6%) patients in the placebo arm with a CR at baseline died due to TEAEs (Table S2A).

Across the measurable, nonmeasurable but evaluable, and no disease subgroups, the relative risks of any‐grade TEAEs of key interest were higher with rucaparib versus placebo, with no differences in risk observed between the subgroups (Figure 4B). Anemia and/or decreased hemoglobin was the only TEAE in which the relative risk of a grade ≥3 event was significantly higher with rucaparib versus placebo across the three subgroups. The incidence of dose modifications in the rucaparib arm ranged from 66.0% to 78.4% across groups, and the incidence of discontinuations ranged from 14.7% to 20.6% (Table S2B). Excluding progression, 5/141 (3.5%) rucaparib‐treated patients with measurable disease at baseline, 1/129 (0.8%) rucaparib‐treated patients with no disease at baseline, and 1/67 (1.5%) patients in the placebo arm with no disease baseline died due to TEAEs (Table S2B).

## 4 DISCUSSION

Understanding how clinical factors may or may not impact the magnitude of clinical benefit can help inform physicians and patients when making decisions about maintenance treatment. These subgroup analyses of data from ARIEL3 show that rucaparib provided PFS benefit over placebo across all prespecified, nested cohorts, irrespective of best response to last platinum‐based chemotherapy or baseline disease status. In both analyses, similar safety profiles were observed between subgroups. Median PFS (investigator‐assessed) with rucaparib maintenance treatment (10.8 months) was significantly longer compared with placebo (5.4 months; HR, 0.36; 95% CI, 0.30–0.45; *p* < 0.0001) in the ARIEL3 overall ITT population.^14^ The reduction in the risk of disease progression among patients in the ITT populations of each subgroup analysis was consistent with that of the overall ARIEL3 ITT population. Similarly, in the nested *BRCA*‐mutated and HRD cohorts within each subgroup analysis, the decreased risk of progression within subgroups was consistent with that of the respective cohorts in the overall ARIEL3 study. These findings expand on prior ARIEL3 analyses, including a new analysis of the subgroups of patients defined by disease status at baseline, which better demonstrates the full spectrum of efficacy for rucaparib among these patients.

Although rucaparib was associated with significantly longer PFS compared with placebo across all subgroups in all analysis cohorts, median PFS with rucaparib was generally longest in the subgroups of patients with a CR, those with no disease, and those with disease that was nonmeasurable but evaluable, regardless of analysis cohort. The PFS extension observed with rucaparib maintenance treatment in patients with a CR and/or no disease is particularly meaningful because it highlights the benefit of maintenance treatment in patients who have had a favorable response to chemotherapy. Our findings clearly demonstrate the usefulness of rucaparib maintenance in patients with a CR to second‐line or later platinum therapy, those with no disease, or those with nonmeasurable disease, because these patients remained progression free according to investigator assessment for up to threefold longer than patients in the placebo arm (equivalent to active surveillance). Furthermore, patients with a CR who did not receive rucaparib maintenance had shorter PFS than patients with a PR who were treated with rucaparib. Although some patients may wish to postpone further therapy if they are not experiencing disease‐related symptoms,^5^ and others may feel that receiving maintenance treatment does not make them a ‘cancer survivor’,^16^ these data indicate the value of maintenance treatment with rucaparib in delaying progression of their disease.

Data from the first‐line setting further justify the use of maintenance treatment in patients with a CR. Women with newly diagnosed ovarian cancer who received olaparib or niraparib maintenance following a CR to first‐line platinum therapy had longer PFS than those who received placebo.^17^, ^18^ In subgroup analyses of women with newly diagnosed ovarian carcinoma who received bevacizumab plus olaparib as maintenance, those with a CR and/or no evidence of disease following first‐line chemotherapy also had longer PFS than those who received only bevacizumab maintenance.^19^

Our analysis also demonstrates that rucaparib maintenance treatment contributed to additional reduction in disease burden for a subset of patients who had measurable disease at baseline or who had nonmeasurable but evaluable disease (i.e., evidence of nontarget lesions per RECIST v1.1) at baseline. Responses observed in patients receiving placebo may be attributed to the sustained cytotoxic effects of immediate prior chemotherapy. In a previous analysis of ARIEL3 data, rucaparib was also shown to provide benefit to patients with measurable disease at baseline (resulting in an objective response rate of 18.4% [26/141] vs. 7.6% [5/66] for placebo in the ITT population).^14^ Our current analysis extends these findings and shows that almost a quarter of patients who had nonmeasurable disease according to RECIST v1.1 (e.g., lesions <10 mm, ascites, pleural or pericardial effusion) experienced a CR with rucaparib maintenance treatment. This deepening of response with rucaparib maintenance treatment may further delay disease progression.

The safety profile for rucaparib‐treated patients in each subgroup was consistent with the profile previously reported for the overall safety population.^14^, ^15^ For patients receiving rucaparib, the pattern of most frequent TEAEs and TEAEs leading to dose interruption/reduction or discontinuation was similar between subgroups. Furthermore, the relative risk of any‐grade and grade ≥3 TEAEs of key interest with rucaparib versus placebo was similar between subgroups, indicating that there are no specific safety concerns with rucaparib for any of the subgroup populations.

Our current analyses are consistent with similar subgroup analyses of niraparib and olaparib as maintenance treatment for recurrent ovarian carcinoma.^10^, ^11^, ^12^ In NOVA, niraparib maintenance treatment provided PFS benefit versus placebo in patients with a germline *BRCA* mutation who had a CR (HR, 0.30; 95% CI, 0.160–0.546; *p* < 0.0001) and those with a PR (HR, 0.24; 95% CI, 0.131–0.441; *p* < 0.0001); benefit was also observed in patients with no germline *BRCA* mutation who had a CR (HR, 0.58; 95% CI, 0.383–0.868; *p* = 0.0082) and those with a PR (HR, 0.35; 95% CI, 0.230–0.532; *p* < 0.0001).^10^ In Study 19, patients with a CR and those with a PR both had a reduced risk of progression with olaparib maintenance treatment versus placebo.^12^

A noteworthy aspect of the ARIEL3 study design was that patients were required to have had measurable disease or CA‐125 >2 times the upper limit of normal at the start of their most recent chemotherapy. Additionally, ARIEL3 allowed patients with bulky residual disease (defined as any lesion >2 cm) to enroll. Therefore, the overall study results and these current subgroup analyses demonstrate that rucaparib maintenance treatment provides clinical benefit for patients who had a clear disease burden at the outset of their prior chemotherapy.

Analyses by best response and measurable disease at baseline were prespecified; however, ARIEL3 was not designed to provide statistical power when evaluating PFS in these exploratory subgroups. A slight discrepancy in the number of patients with a CR (*n* = 126) and the number of patients with no disease (*n* = 130) may be considered another limitation of the current analyses since the number of patients in these two subgroups would be expected to be the same. This discrepancy arose due to differences in the way the data for response to prior therapy and baseline disease were reported by investigators: the former were entered into the interactive web and voice response system at the time of randomization, whereas the latter were recorded in patients’ electronic case report forms and verified against source data (i.e., patient scans).

In conclusion, these results from ARIEL3 demonstrate the broad efficacy and safety of maintenance treatment with rucaparib in patients with disease present at baseline, whether or not disease was measurable, and in patients with no disease and/or a CR following their most recent platinum‐based chemotherapy. Patients from all of the subgroups examined here should be considered for rucaparib maintenance treatment, as all subgroups derived similar benefit from rucaparib compared with placebo, which would be equivalent to active surveillance in clinical practice.

## CONFLICT OF INTEREST

A.O. has served on advisory boards for Clovis Oncology, AstraZeneca, Deciphera, Genmab/Seattle Genetics, GlaxoSmithKline/Tesaro, ImmunoGen, Merck/Merck Sharp & Dohme, Mersana Therapeutics, PharmaMar, and Roche; has received support for travel or accommodation from Clovis Oncology, AstraZeneca, PharmaMar, and Roche; and reports institutional research grant support from Clovis Oncology, AbbVie Deutschland, Ability Pharmaceuticals, Advaxis, Aeterna Zentaris, Amgen, Aprea Therapeutics, Eisai, ImmunoGen, Merck/Merck Sharp & Dohme, Millennium Pharmaceuticals, PharmaMar, Roche, and Tesaro. A.M.O. reports grants to his institution from AstraZeneca; has served on steering committees for Clovis Oncology and AstraZeneca (uncompensated); has served in an advisory role for AstraZeneca and GlaxoSmithKline (uncompensated); and has acted as a principal investigator on investigator‐initiated trials with agents from Clovis Oncology, AstraZeneca, and GlaxoSmithKline. D.L. has served in a consulting or advisory role for Clovis Oncology, AstraZeneca, Genmab, ImmunoGen, Merck, PharmaMar, Roche, Takeda, and Tesaro/GlaxoSmithKline, has received institutional research support from Merck, PharmaMar, and Tesaro/GlaxoSmithKline and received support for travel or accommodation from AstraZeneca, PharmaMar, Roche, and Tesaro/GlaxoSmithKline. C.A. has served on a steering committee for AbbVie and Genentech; served on advisory boards for Clovis Oncology, AbbVie, Eisai/Merck, ImmunoGen, Mersana Therapeutics, Roche, and Tesaro; and received research grants from Clovis Oncology, AbbVie, AstraZeneca, and Genentech. A.D. has served in a consulting or advisory role for Precision Oncology Australia, Shire Pharmaceuticals, and Specialised Therapeutics Australia. N.C. has served in a consulting or advisory role for Clovis Oncology, Advaxis, AstraZeneca, BIOCAD, GlaxoSmithKline, Merck Sharp & Dohme, Pfizer, PharmaMar, Roche, Takeda, and Tesaro. J.I.W. has received research support from AbbVie and AstraZeneca and served on advisory boards for AstraZeneca. A.R.C. has served in a consulting or advisory role for AstraZeneca, GlaxoSmithKline, and Roche; received research funding from AstraZeneca; and received support for travel and accommodation for congress attendance from Clovis Oncology, AstraZeneca, and GlaxoSmithKline. G.S. has served in a consulting or advisory role for Clovis Oncology, AstraZeneca, PharmaMar, Roche, and Tesaro. A.L. has served on advisory boards for Clovis Oncology, Ability Pharmaceuticals, AstraZeneca, BIOCAD, GamaMabs, Genmab/Seattle Genetics, Gritstone, GlaxoSmithKline, Merck Serono, Merck Sharp & Dohme, and Tesaro; steering committee for Merck Sharp & Dohme; reports institutional support for clinical trials or academic research from Clovis Oncology, Ability Pharmaceuticals, Agenus, AstraZeneca, Incyte, Inivata, Iovance, Merck Sharp & Dohme, Pfizer, Roche, Sanofi, and Tesaro; and reports boarding and travel expenses for congress activities from Clovis Oncology, AstraZeneca, and Roche. R.W.H. has served on speakers’ bureaus for Clovis Oncology, AstraZeneca, and Tesaro, and on advisory boards for Clovis Oncology and AstraZeneca. M.A.G. has served on speakers’ bureaus for Clovis Oncology, AstraZeneca, PharmaMar, and Roche. P.C.F. has served on advisory boards for Clovis Oncology and AstraZeneca and received honoraria from AstraZeneca. J.C.G. has received honoraria from AstraZeneca and Bristol‐Myers Squibb; served in a consulting or advisory role for AstraZeneca, Bristol‐Myers Squibb, GlaxoSmithKline, Merck Sharp & Dohme, and Tesaro; served on speakers’ bureaus for AstraZeneca, Ipsen, and Merck Sharp & Dohme; and received support for travel and/or accommodation from Astellas and AstraZeneca. D.M.O. has served on advisory boards for Clovis Oncology, AbbVie, AstraZeneca, Eisai, Genentech/Roche, Genelux, Iovance Biotherapeutics, Janssen, Novocure, Regeneron, and Tesaro; has served on steering committees for Clovis Oncology, Agenus, Amgen, and Novocure; has served as a consultant for AbbVie, Ambry, AstraZeneca, Genentech/Roche, Gynecologic Oncology Group Foundation, and Tesaro; has given a presentation on ovarian cancer at the National Comprehensive Cancer Network; and his institution has received research support from Clovis Oncology, AbbVie, Agenus, Ajinomoto, Amgen, Array BioPharma, AstraZeneca, Bristol‐Myers Squibb, Cerulean Pharma, Eisai, EMD Serono, ERGOMED Clinical Research, Genentech, Gynecologic Oncology Group, INC Research, inVentiv Health Clinical, Iovance Biotherapeutics, Janssen Research and Development, Ludwig Institute for Cancer Research, New Mexico Cancer Care Alliance, Novocure, PRA International, Regeneron Pharmaceuticals, Serono, Stemcentrx, Tesaro, TRACON Pharmaceuticals, VentiRx, and Yale University. D.K.A. has served as a scientific advisor for Morphotek and received research funding from Clovis Oncology, Advaxis, AstraZeneca, Pfizer, Syndax, and Tesaro. S.B. has served on advisory boards and received honoraria from Clovis Oncology, AstraZeneca, Genmab, GlaxoSmithKline, Immunogen, Merck Sereno, Merck Sharp & Dohme, Mersana, Pfizer, Roche, Seattle Genetics, and Tesaro; received honoraria for lectures from AstraZeneca/Merck Sharp & Dohme, GlaxoSmithKline, Pfizer, Roche, and Tesaro; received support for travel or accommodation from NuCana and Tesaro; and reports institutional funding from AstraZeneca, GlaxoSmithKline, and Tesaro. J.G.‐D. has received research funding from AstraZeneca, Pierre Fabre, and Pfizer; received personal fees from Clovis Oncology, Astellas, Pierre Fabre, and Pfizer; and received nonfinancial support from Astellas, Pierre Fabre, and Pfizer. E.M.S. has nothing to disclose. T.C., L.M., and S.G. are employees of Clovis Oncology and may own stock or have stock options in that company. J.A.L. has received lecture fees from Clovis Oncology, AstraZeneca, and Pfizer; served on advisory boards for Clovis Oncology, Artios Pharma, AstraZeneca, Cristal Therapeutics, Merck/Merck Sharp & Dohme, Pfizer, Regeneron, Roche, Seattle Genetics, and Tesaro; and received research grants from AstraZeneca and Merck/Merck Sharp & Dohme. R.L.C. reports grants from Clovis Oncology, AstraZeneca, Gateway Foundation, Janssen, Judy Reis/Albert Pisani, MD, Ovarian Cancer Research Fund, Merck, National Institutes of Health, Roche/Genentech, and V‐Foundation; has served as an advisor to Clovis Oncology, Agenus, AstraZeneca, GamaMabs, Genmab, Janssen, OncoQuest, Pfizer (Medivation), Regeneron, Roche/Genentech, and Tesaro; and has an endowment as the Ann Rife Cox Chair in Gynecology.

## ETHICAL APPROVAL

The study was approved by national or local institutional review boards and performed in accordance with the Declaration of Helsinki and Good Clinical Practice Guidelines of the International Council for Harmonisation.

## Supporting information

Table S1–S2

## Data Availability

Requests for de‐identified datasets for the results reported in this publication will be made available to qualified researchers following submission of a methodologically sound proposal to medinfo@clovisoncology.com. Data will be made available for such requests following online publication of this article and for 1 year thereafter in compliance with applicable privacy laws, data protection, and requirements for consent and anonymization. Data will be provided by Clovis Oncology. The redacted protocol for the ARIEL3 clinical study is available on ClinicalTrials.gov (NCT01968213). Clovis Oncology does not share identified participant data or a data dictionary.
