# Supplementary material for: Maintenance treatment with rucaparib for recurrent ovarian carcinoma in ARIEL3, a randomized phase 3 trial: The effects of best response to last platinum‐based regimen and disease at baseline on efficacy and safety
Source: Cancer Med. 2021 Sep 21;10(20):7162–73. doi: 10.1002/cam4.4260 (PMC8525125; doi:10.1002/cam4.4260)

SUPPORTING INFORMATION

METHODS

## Study Design and Patients

Eligible patients were aged ≥18 years, had platinum-sensitive, high-grade serous or endometrioid ovarian, primary peritoneal, or fallopian tube carcinoma, had received two or more previous platinum-based chemotherapy regimens, had a baseline cancer antigen 125 (CA-125) measurement below the upper limit of normal, and must have achieved either a CR according to Response Evaluation Criteria in Solid Tumors (RECIST) v1.1,^1^ or a partial response (PR) defined either according to RECIST v1.1 or as a serological response based on Gynecologic Cancer InterGroup CA-125 response criteria^2^ to their last platinum-based regimen. Patients had to have measurable disease or CA-125 >2 times the upper limit of normal immediately before their most recent chemotherapy regimen, to assess response to their last platinum-based regimen.^2^ To ensure tumor response was driven by a patient’s most recent chemotherapy and not secondary cytoreduction, surgery resulting in complete gross resection was not permitted as a component of the most recent treatment regimen.

## Procedures

Screening procedures were described previously.^3^ Patients were stratified based on homologous recombination repair gene mutation status (based on gene mutation only; mutation in *BRCA1* or *BRCA2*, mutation in a non-*BRCA* gene associated with homologous recombination, or no mutation in *BRCA* or a homologous recombination gene), progression-free interval following penultimate platinum-based regimen (6-12 months or >12 months), and best response to most recent platinum-based regimen (complete response [CR] or PR), and then randomized 2:1 to receive oral rucaparib 600 mg twice daily or placebo. Disease assessments were conducted at screening, every 12 weeks during treatment (and after treatment for patients who discontinued for reasons other than disease progression), following clinical symptoms, and at treatment discontinuation. Details of study drug administration and dosing were described previously.^3^

Patients received oral rucaparib 600 mg twice daily or placebo in continuous 28-day cycles until disease progression (assessed per RECIST v1.1), death, or other reasons for discontinuation. Dose reductions (in decrements of 120 mg down to 240 mg) were permitted if a patient had a grade ≥3 or persistent grade 2 adverse event. Treatment was discontinued following toxicity-related treatment interruption of ˃14 consecutive days.

Safety was assessed by monitoring for treatment-emergent adverse events classified per the Medical Dictionary for Drug Regulatory Activities, version 19.1,^4^ and graded as per the National Cancer Institute Common Terminology Criteria for Adverse Events, version 4.03.^5^

## Statistical Analyses

Progression-free survival was evaluated using Kaplan-Meier methodology; patients without documented progression were censored as of their last tumor assessment. A stratified log-rank test that included the randomization strata was used to compare treatments. Additionally, a stratified Cox proportional hazards model was used to calculate the hazard ratio between the treatment arms for progression-free survival. The subgroup efficacy endpoint analyses presented in this manuscript were tested at a one-sided 0.025 significance level, without any multiplicity adjustment.

For safety analyses, risk differences with 95% confidence intervals were summarized for specific treatment-emergent adverse events. Risk difference was defined as the difference in percentage between the rucaparib and the placebo arms. Confidence intervals were estimated based on normal distribution assumption.

# References

1. Eisenhauer EA, Therasse P, Bogaerts J, et al. New response evaluation criteria in solid tumours: revised RECIST guideline (version 1.1). *Eur J Cancer.* 2009;45:228-247.

2. Rustin GJ, Vergote I, Eisenhauer E, et al. Definitions for response and progression in ovarian cancer clinical trials incorporating RECIST 1.1 and CA 125 agreed by the Gynecological Cancer Intergroup (GCIG). *Int J Gynecol Cancer.* 2011;21:419-423.

3. Coleman RL, Oza AM, Lorusso D, et al. Rucaparib maintenance treatment for recurrent ovarian carcinoma after response to platinum therapy (ARIEL3): a randomised, double-blind, placebo-controlled, phase 3 trial. *Lancet.* 2017;390:1949-1961.

4. Brown EG, Wood L, Wood S. The medical dictionary for regulatory activities (MedDRA). *Drug Saf.* 1999;20:109-117.

5. National Cancer Institute. NCI Term Browser, CTCAE. 2010; <https://nciterms.nci.nih.gov/ncitbrowser/pages/vocabulary.jsf?dictionary=CTCAE&version=4.03>. Accessed May 7, 2021.

**Table S1.** Baseline Patient Demographics, Disease Characteristics, and Prior Therapies According to (A) Best Response to Last Platinum-Based Chemotherapy and (B) Disease at Baseline

**A**

| **Characteristic** | **Complete response^a^** | | **Partial response^b^** | |
| --- | --- | --- | --- | --- |
|  | **Rucaparib (n = 126)** | **Placebo (n = 64)** | **Rucaparib  (n = 249)** | **Placebo (n = 125)** |
| Median age, years (range) | 58 (42-83) | 60 (41-85) | 62 (39-84) | 63 (36-84) |
| Diagnosis, n (%) |  |  |  |  |
| Epithelial ovarian cancer | 110 (87.3) | 53 (82.8) | 202 (81.1) | 106 (84.8) |
| Fallopian tube cancer | 7 (5.6) | 6 (9.4) | 25 (10.0) | 4 (3.2) |
| Primary peritoneal cancer | 9 (7.1) | 5 (7.8) | 22 (8.8) | 14 (11.2) |
| Serous adenocarcinoma | 0 | 0 | 0 | 1 (0.8)^c^ |
| Histology, n (%) |  |  |  |  |
| Serous | 120 (95.2) | 59 (92.2) | 237 (95.2) | 120 (96.0) |
| Endometrioid | 5 (4.0) | 5 (7.8) | 11 (4.4) | 2 (1.6) |
| Other or mixed | 1 (0.8) | 0 | 1 (0.4) | 3 (2.4) |
| Bulky disease per independent radiological review, n (%) |  |  |  |  |
| Yes^d^ | 18 (14.3) | 3 (4.7) | 53 (21.3) | 26 (20.8) |
| No^e^ | 108 (85.7) | 61 (95.3) | 196 (78.7) | 99 (79.2) |
| *BRCA* and LOH status, n (%) |  |  |  |  |
| *BRCA* mutated | 43 (34.1) | 22 (34.4) | 87 (34.9) | 44 (35.2) |
| *BRCA* wild type | 83 (65.9) | 42 (65.6) | 162 (65.1) | 81 (64.8) |
| LOH high | 39 (31.0) | 19 (29.7) | 67 (26.9) | 33 (26.4) |
| LOH low | 36 (28.6) | 20 (31.3) | 71 (28.5) | 34 (27.2) |
| LOH indeterminate^f^ | 8 (6.3) | 3 (4.7) | 24 (9.6) | 14 (11.2) |
| ECOG PS, n (%) |  |  |  |  |
| 0 | 99 (78.6) | 53 (82.8) | 181 (72.7) | 83 (66.4) |
| 1 | 27 (21.4) | 11 (17.2) | 68 (27.3) | 42 (33.6) |
| Median no. of prior chemotherapy regimens (range) | 2 (2-5) | 2 (2-5) | 2 (2-6) | 2 (2-6) |
| 2, n (%) | 76 (60.3) | 42 (65.6) | 155 (62.2) | 82 (65.6) |
| 3, n (%) | 36 (28.6) | 15 (23.4) | 72 (28.9) | 27 (21.6) |
| ≥4, n (%) | 14 (11.1) | 7 (10.9) | 22 (8.8) | 16 (12.8) |
| Previous bevacizumab use, n (%) | 30 (23.8) | 11 (17.2) | 53 (21.3) | 32 (25.6) |
| Median time to progression with penultimate platinum, months (range) | 14.4 (6.0-79.4) | 15.6 (7.3-238.5) | 13.5 (5.8-120.0) | 14.3 (6.0-76.6) |
| 6 to ≤12 months, n (%) | 44 (34.9) | 23 (35.9) | 107 (43.0) | 53 (42.4) |
| >12 months, n (%) | 82 (65.1) | 41 (64.1) | 142 (57.0) | 72 (57.6) |
| Abbreviations: CA-125, cancer antigen 125; ECOG PS, Eastern Cooperative Oncology Group performance status; GCIG, Gynecologic Cancer InterGroup; LOH, loss of heterozygosity; RECIST v1.1, Response Evaluation Criteria in Solid Tumors version 1.1.  ^a^ Per RECIST v1.1.  ^b^ Per RECIST v1.1 or serological response per GCIG CA-125 criteria.  ^c^ Fallopian and/or ovarian in origin.  ^d^ Bulky residual disease was defined as any lesion >2 cm.  ^e^ No bulky residual disease was defined as no disease or all lesions ≤2 cm.  ^f^ Tumor sample was not evaluable for percentage of genomic LOH due to low tumor content or low aneuploidy. | | | | |

**B**

| **Characteristic** | **Measurable disease** | | **Nonmeasurable disease** | | **No disease** | |
| --- | --- | --- | --- | --- | --- | --- |
|  | **Rucaparib (n = 141)** | **Placebo (n = 66)** | **Rucaparib (n = 104)** | **Placebo (n = 56)** | **Rucaparib (n = 130)** | **Placebo (n = 67)** |
| Median age, years (range) | 61 (39-84) | 65 (40-84) | 63 (43-78) | 62 (41-78) | 58 (42-83) | 60 (36-85) |
| Diagnosis, n (%) |  |  |  |  |  |  |
| Epithelial ovarian cancer | 119 (84.4) | 56 (84.8) | 83 (79.8) | 43 (76.8) | 110 (84.6) | 60 (89.6) |
| Fallopian tube cancer | 13 (9.2) | 5 (7.6) | 10 (9.6) | 2 (3.6) | 9 (6.9) | 3 (4.5) |
| Primary peritoneal cancer | 9 (6.4) | 4 (6.1) | 11 (10.6) | 11 (19.6) | 11 (8.5) | 4 (6.0) |
| Serous adenocarcinoma | 0 | 1 (1.5)^a^ | 0 | 0 | 0 | 0 |
| Histology, n (%) |  |  |  |  |  |  |
| Serous | 133 (94.3) | 63 (95.5) | 101 (97.1) | 55 (98.2) | 123 (94.6) | 61 (91.0) |
| Endometrioid | 7 (5.0) | 1 (1.5) | 3 (2.9) | 0 | 6 (4.6) | 6 (9.0) |
| Other or mixed | 1 (0.7) | 2 (3.0) | 0 | 1 (1.8) | 1 (0.8) | 0 |
| Bulky disease per independent radiological review, n (%) |  |  |  |  |  |  |
| Yes^b^ | 43 (30.5) | 22 (33.3) | 16 (15.4) | 4 (7.1) | 12 (9.2) | 3 (4.5) |
| No^c^ | 98 (69.5) | 44 (66.7) | 88 (84.6) | 52 (92.9) | 118 (90.8) | 64 (95.5) |
| *BRCA* and LOH status, n (%) | | | | | | |
| *BRCA* mutated | 40 (28.4) | 23 (34.8) | 38 (36.5) | 16 (28.6) | 52 (40.0) | 27 (40.3) |
| *BRCA* wild type | 101 (71.6) | 43 (65.2) | 66 (63.5) | 40 (71.4) | 78 (60.0) | 40 (59.7) |
| LOH high | 45 (31.9) | 18 (27.3) | 28 (26.9) | 14 (25.0) | 33 (25.4) | 20 (29.9) |
| LOH low | 45 (31.9) | 21 (31.8) | 26 (25.0) | 16 (28.6) | 36 (27.7) | 17 (25.4) |
| LOH indeterminate^d^ | 11 (7.8) | 4 (6.1) | 12 (11.5) | 10 (17.9) | 9 (6.9) | 3 (4.5) |
| ECOG PS, n (%) |  |  |  |  |  |  |
| 0 | 100 (70.9) | 42 (63.6) | 77 (74.0) | 41 (73.2) | 103 (79.2) | 53 (79.1) |
| 1 | 41 (29.1) | 24 (36.4) | 27 (26.0) | 15 (26.8) | 27 (20.8) | 14 (20.9) |
| Median no. of prior chemotherapy regimens (range) | 2 (2-6) | 2 (2-5) | 2 (2-6) | 2 (2-6) | 2 (2-5) | 2 (2-5) |
| 2, n (%) | 86 (61.0) | 49 (74.2) | 64 (61.5) | 35 (62.5) | 81 (62.3) | 40 (59.7) |
| 3, n (%) | 43 (30.5) | 10 (15.2) | 30 (28.8) | 13 (23.2) | 35 (26.9) | 19 (28.4) |
| ≥4, n (%) | 12 (8.5) | 7 (10.6) | 10 (9.6) | 8 (14.3) | 14 (10.8) | 8 (11.9) |
| Previous bevacizumab use, n (%) | 29 (20.6) | 16 (24.2) | 24 (23.1) | 12 (21.4) | 30 (23.1) | 15 (22.4) |
| Median time to progression with penultimate platinum, months (range) | 13.5  (5.8-115.4) | 15.9  (6.4-71.6) | 12.8  (6.1-120.0) | 12.0  (6.0-76.6) | 14.9  (6.0-79.4) | 16.4  (6.4-238.5) |
| 6 to ≤12 months, n (%) | 65 (46.1) | 21 (31.8) | 47 (45.2) | 28 (50.0) | 39 (30.0) | 27 (40.3) |
| >12 months, n (%) | 76 (53.9) | 45 (68.2) | 57 (54.8) | 28 (50.0) | 91 (70.0) | 40 (59.7) |
| Response to last platinum, n (%) | | | | | | |
| CR per RECIST v1.1 | 8 (5.7) | 7 (10.6) | 13 (12.5) | 5 (8.9) | 105 (80.8) | 52 (77.6) |
| PR per RECIST v1.1 or serological response per GCIG CA-125 criteria | 133 (94.3) | 59 (89.4) | 91 (87.5) | 51 (91.1) | 25 (19.2) | 15 (22.4) |
| Abbreviations: CA-125, cancer antigen 125; CR, complete response; ECOG PS, Eastern Cooperative Oncology Group performance status; GCIG, Gynecologic Cancer InterGroup; LOH, loss of heterozygosity; PR, partial response; RECIST v1.1, Response Evaluation Criteria in Solid Tumors version 1.1.  ^a^ Fallopian and/or ovarian in origin.  ^b^ Bulky residual disease was defined as any lesion >2 cm.  ^c^ No bulky residual disease was defined as no disease or all lesions ≤2 cm.  ^d^ Tumor sample was not evaluable for percentage of genomic LOH due to low tumor content or low aneuploidy. | | | | | | |

**Table S2.** Summary of Safety and TEAEs in Subgroups. Subgroups Defined by (A) Best Response to Last Platinum-based Chemotherapy Regimen and (B) Disease at Baseline

**A**

|  | **Complete response*,* n (%)^a^** | | | | **Partial response, n (%)^b^** | | | |
| --- | --- | --- | --- | --- | --- | --- | --- | --- |
|  | **Rucaparib (n = 125)^c^** | | **Placebo (n = 64)** | | **Rucaparib (n = 247)^c^** | | **Placebo (n = 125)** | |
| Overall TEAEs (any grade) | 125 (100) | | 61 (95.3) | | 247 (100) | | 121 (96.8) | |
| All grade ≥3 | 78 (62.4) | | 12 (18.8) | | 153 (61.9) | | 19 (15.2) | |
| Treatment interruption and/or dose reduction due to a TEAE | 96 (76.8) | | 12 (18.8) | | 175 (70.9) | | 8 (6.4) | |
| Treatment interruption | 85 (68.0) | | 11 (17.2) | | 163 (66.0) | | 8 (6.4) | |
| Dose reduction | 75 (60.0) | | 5 (7.8) | | 134 (54.3) | | 3 (2.4) | |
| Discontinuation due to a TEAE^d^ | 18 (14.4) | | 1 (1.6) | | 46 (18.6) | | 2 (1.6) | |
| Deaths related to a TEAE | 1 (0.8) | | 1 (1.6) | | 7 (2.8) | | 1 (0.8) | |
| Deaths due disease progression | 0 | | 0 | | 2 (0.8) | | 1 (0.8) | |
| Deaths due to nonprogression events | 1 (0.8)^e^ | | 1 (1.6)^f^ | | 5 (2.0)^g^ | | 0 | |
| **Individual TEAEs occurring in ≥20% patients, n (%)^h^** | **Any grade** | **Grade ≥3** | **Any grade** | **Grade ≥3** | **Any grade** | **Grade ≥3** | **Any grade** | **Grade ≥3** |
| Nausea | 88 (70.4) | 5 (4.0) | 23 (35.9) | 0 | 196 (79.4) | 9 (3.6) | 47 (37.6) | 1 (0.8) |
| Asthenia/fatigue | 96 (76.8) | 13 (10.4) | 26 (40.6) | 2 (3.1) | 171 (69.2) | 16 (6.5) | 59 (47.2) | 3 (2.4) |
| Anemia/decreased hemoglobin | 49 (39.2) | 30 (24.0) | 1 (1.6) | 0 | 98 (39.7) | 53 (21.5) | 8 (6.4) | 1 (0.8) |
| Constipation | 43 (34.4) | 1 (0.8) | 14 (21.9) | 1 (1.6) | 97 (39.3) | 6 (2.4) | 30 (24.0) | 1 (0.8) |
| Dysgeusia | 51 (40.8) | 0 | 2 (3.1) | 0 | 97 (39.3) | 0 | 11 (8.8) | 0 |
| Vomiting | 46 (36.8) | 7 (5.6) | 5 (7.8) | 0 | 93 (37.7) | 9 (3.6) | 24 (19.2) | 2 (1.6) |
| Diarrhea | 47 (37.6) | 2 (1.6) | 10 (15.6) | 0 | 82 (33.2) | 1 (0.4) | 33 (26.4) | 2 (1.6) |
| ALT/AST elevation | 55 (44.0) | 20 (16.0) | 1 (1.6) | 0 | 78 (31.6) | 19 (7.7) | 5 (4.0) | 0 |
| Abdominal pain | 44 (35.2) | 4 (3.2) | 18 (28.1) | 0 | 76 (30.8) | 8 (3.2) | 32 (25.6) | 1 (0.8) |
| Thrombocytopenia/decreased platelet count | 37 (29.6) | 6 (4.8) | 3 (4.7) | 0 | 74 (30.0) | 15 (6.1) | 2 (1.6) | 0 |
| Decreased appetite | 29 (23.2) | 1 (0.8) | 9 (14.1) | 0 | 65 (26.3) | 2 (0.8) | 16 (12.8) | 0 |
| Neutropenia/decreased neutrophil count | 27 (21.6) | 11 (8.8) | 1 (1.6) | 0 | 49 (19.8) | 21 (8.5) | 8 (6.4) | 2 (1.6) |
| Headache | 33 (26.4) | 1 (0.8) | 10 (15.6) | 0 | 40 (16.2) | 0 | 21 (16.8) | 1 (0.8) |
| Abbreviations: ALT, alanine aminotransferase; AST, aspartate aminotransferase; CA-125, cancer antigen 125; GCIG, Gynecologic Cancer InterGroup; RECIST v1.1, Response Evaluation Criteria in Solid Tumors version 1.1; TEAE, treatment-emergent adverse event.  ^a^ Per RECIST v1.1.  ^b^ Per RECIST v1.1 or serological response per GCIG CA-125 criteria.  ^c^ Three patients randomized to the rucaparib arm did not receive a dose of rucaparib and are excluded from the safety population.  ^d^ Excluding disease progression.  ^e^ Cardiac arrest.  ^f^ Pulmonary embolism.  ^g^ Acute myeloid leukemia (n = 1), hematophagic histiocytosis (n = 1), high-grade B-cell unclassifiable lymphoma (n = 1), myelodysplastic syndrome (n = 2).  ^h^ Sorted by decreasing incidence of any-grade TEAEs in rucaparib-treated patients who had a partial response to last platinum-based therapy. | | | | | | | | |

**B**

|  | **Measurable disease** | | | | **Nonmeasurable disease** | | | | **No disease** | | | |
| --- | --- | --- | --- | --- | --- | --- | --- | --- | --- | --- | --- | --- |
|  | **Rucaparib (n = 141)^a^** | | **Placebo (n = 66)** | | **Rucaparib (n = 102)^a^** | | **Placebo (n = 56)** | | **Rucaparib (n = 129)^a^** | | **Placebo (n = 67)** | |
| Overall TEAEs (any grade) | 141 (100) | | 63 (95.5) | | 102 (100) | | 54 (96.4) | | 129 (100) | | 65 (97.0) | |
| All grade ≥3 | 84 (59.6) | | 9 (13.6) | | 68 (66.7) | | 10 (17.9) | | 79 (61.2) | | 12 (17.9) | |
| Treatment interruption and/or dose reduction due to a TEAE | 93 (66.0) | | 3 (4.5) | | 80 (78.4) | | 4 (7.1) | | 98 (76.0) | | 13 (19.4) | |
| Treatment interruption | 83 (58.9) | | 3 (4.5) | | 77 (75.5) | | 4 (7.1) | | 88 (68.2) | | 12 (17.9) | |
| Dose reduction | 72 (51.1) | | 2 (3.0) | | 62 (60.8) | | 2 (3.6) | | 75 (58.1) | | 4 (6.0) | |
| Discontinuation due to a TEAE^b^ | 24 (17.0) | | 1 (1.5) | | 21 (20.6) | | 2 (3.6) | | 19 (14.7) | | 0 | |
| Deaths related to a TEAE | 7 (5.0) | | 0 | | 0 | | 1 (1.8) | | 1 (0.8) | | 1 (1.5) | |
| Deaths due disease progression | 2 (1.4) | | 0 | | 0 | | 1 (1.8) | | 0 | | 0 | |
| Deaths due to nonprogression events | 5 (3.5)^c^ | | 0 | | 0 | | 0 | | 1 (0.8)^d^ | | 1 (1.5)^e^ | |
| **Individual TEAEs occurring in ≥20% patients, n (%)^f^** | **Any grade** | **Grade ≥3** | **Any grade** | **Grade ≥3** | **Any grade** | **Grade ≥3** | **Any grade** | **Grade ≥3** | **Any grade** | **Grade ≥3** | **Any grade** | **Grade ≥3** |
| Nausea | 108 (76.6) | 6 (4.3) | 23 (34.8) | 0 | 79 (77.5) | 3 (2.9) | 23 (41.1) | 1 (1.8) | 97 (75.2) | 5 (3.9) | 24 (35.8) | 0 |
| Asthenia/fatigue | 101 (71.6) | 8 (5.7) | 30 (45.5) | 2 (3.0) | 70 (68.6) | 9 (8.8) | 26 (46.4) | 2 (3.6) | 96 (74.4) | 12 (9.3) | 29 (43.3) | 1 (1.5) |
| Constipation | 58 (41.1) | 2 (1.4) | 14 (21.2) | 1 (1.5) | 40 (39.2) | 3 (2.9) | 12 (21.4) | 0 | 42 (32.6) | 2 (1.6) | 18 (26.9) | 1 (1.5) |
| Dysgeusia | 52 (36.9) | 0 | 8 (12.1) | 0 | 45 (44.1) | 0 | 3 (5.4) | 0 | 51 (39.5) | 0 | 2 (3.0) | 0 |
| Vomiting | 52 (36.9) | 5 (3.5) | 12 (18.2) | 1 (1.5) | 38 (37.3) | 3 (2.9) | 9 (16.1) | 1 (1.8) | 49 (38.0) | 8 (6.2) | 8 (11.9) | 0 |
| Anemia/decreased hemoglobin | 49 (34.8) | 31 (22.0) | 5 (7.6) | 0 | 42 (41.2) | 21 (20.6) | 1 (1.8) | 1 (1.8) | 56 (43.4) | 31 (24.0) | 3 (4.5) | 0 |
| Diarrhea | 47 (33.3) | 1 (0.7) | 19 (28.8) | 2 (3.0) | 35 (34.3) | 0 | 10 (17.9) | 0 | 47 (36.4) | 2 (1.6) | 14 (20.9) | 0 |
| ALT/AST elevation | 45 (31.9) | 8 (5.7) | 3 (4.5) | 0 | 32 (31.4) | 8 (7.8) | 2 (3.6) | 0 | 56 (43.4) | 23 (17.8) | 1 (1.5) | 0 |
| Abdominal pain | 40 (28.4) | 3 (2.1) | 21 (31.8) | 0 | 34 (33.3) | 4 (3.9) | 11 (19.6) | 0 | 46 (35.7) | 5 (3.9) | 18 (26.9) | 1 (1.5) |
| Thrombocytopenia/decreased platelet count | 39 (27.7) | 11 (7.8) | 0 | 0 | 33 (32.4) | 4 (3.9) | 2 (3.6) | 0 | 39 (30.2) | 6 (4.7) | 3 (4.5) | 0 |
| Arthralgia | 30 (21.3) | 1 (0.7) | 11 (16.7) | 0 | 10 (9.8) | 0 | 7 (12.5) | 0 | 26 (20.2) | 1 (0.8) | 6 (9.0) | 0 |
| Neutropenia/decreased neutrophil count | 29 (20.6) | 13 (9.2) | 3 (4.5) | 0 | 20 (19.6) | 7 (6.9) | 4 (7.1) | 2 (3.6) | 27 (20.9) | 12 (9.3) | 2 (3.0) | 0 |
| Headache | 27 (19.1) | 1 (0.7) | 10 (15.2) | 0 | 16 (15.7) | 0 | 9 (16.1) | 1 (1.8) | 30 (23.3) | 0 | 12 (17.9) | 0 |
| Decreased appetite | 24 (17.0) | 0 | 9 (13.6) | 0 | 37 (36.3) | 1 (1.0) | 7 (12.5) | 0 | 33 (25.6) | 2 (1.6) | 9 (13.4) | 0 |
| Dyspepsia | 20 (14.2) | 1 (0.7) | 5 (7.6) | 0 | 10 (9.8) | 0 | 0 | 0 | 27 (20.9) | 0 | 4 (6.0) | 0 |
| Back pain | 18 (12.8) | 0 | 11 (16.7) | 0 | 12 (11.8) | 0 | 9 (16.1) | 0 | 27 (20.9) | 0 | 6 (9.0) | 0 |
| Abbreviations: ALT, alanine aminotransferase; AST, aspartate aminotransferase; TEAE, treatment-emergent adverse event.  ^a^ Three patients randomized to the rucaparib arm did not receive a dose of rucaparib and are excluded from the safety population.  ^b^ Excluding disease progression.  ^c^ Acute myeloid leukemia (n =1), hematophagic histiocytosis (n = 1), high-grade B-cell unclassifiable lymphoma (n = 1), myelodysplastic syndrome (n = 2).  ^d^ Cardiac arrest.  ^e^ Pulmonary embolism.  ^f^ Sorted by decreasing incidence of any-grade TEAEs in rucaparib-treated patients who had measurable disease at baseline. | | | | | | | | | | | | |

**Figure S1.** BICR-assessed PFS according to best response to last platinum-based chemotherapy. Patients with a CR to last platinum-based chemotherapy in the (A) *BRCA*-mutated cohort, (B) HRD cohort, and (C) ITT population. Patients with a PR to last platinum-based chemotherapy in the (D) *BRCA*-mutated cohort, (E) HRD cohort, and (F) ITT population. *p* values were nonsignificant for treatment by best response subgroup (CR vs PR) interaction tests (*BRCA*-mutated cohort, *p* = 0.9192; HRD cohort, *p* = 0.8383; ITT population, *p* = 0.7976). *p* values are presented for descriptive purposes only. BICR indicates blinded independent central review; CI, confidence interval; CR, complete response; HR, hazard ratio; HRD, homologous recombination deficient; ITT, intent to treat; NR, not reached; PFS, progression-free survival; PR, partial response.

**
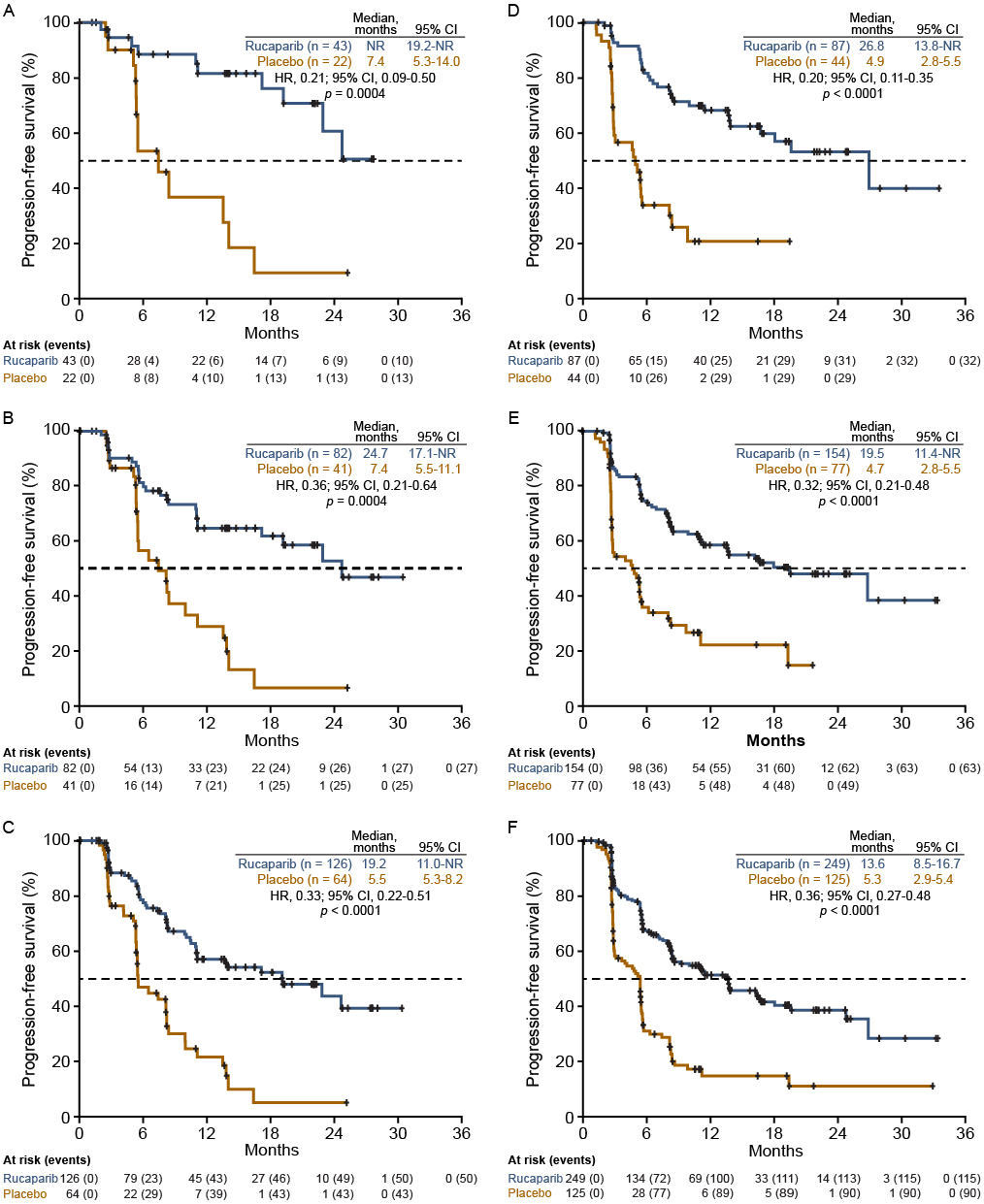
**

**Figure S2.** BICR-assessed PFS according to disease at baseline. Patients with measurable disease at baseline in the (A) *BRCA*-mutated cohort, (B) HRD cohort, and (C) ITT population. Patients with nonmeasurable but evaluable disease at baseline in the (D) *BRCA*-mutated cohort, (E) HRD cohort, and (F) ITT population. Patients with no disease at baseline in the (G) *BRCA*-mutated cohort, (H) HRD cohort, and (I) ITT population. *p* values were nonsignificant for treatment by baseline disease subgroup interaction tests (*BRCA*-mutated cohort: no disease vs nonmeasurable disease, *p* = 0.4098; no disease vs measurable disease, *p* = 0.3768; nonmeasurable disease vs measurable disease, *p* = 0.2243; HRD cohort: no disease vs nonmeasurable disease, *p* = 0.6103; no disease vs measurable disease, *p* = 0.4095; nonmeasurable disease vs measurable disease, *p* = 0.5051; ITT population: no disease vs nonmeasurable disease, *p* = 0.6691; no disease vs measurable disease, *p* = 0.9768; nonmeasurable disease vs measurable disease, *p* = 0.2602). *p* values are presented for descriptive purposes only. BICR indicates blinded independent central review; CI, confidence interval; HR, hazard ratio; HRD, homologous recombination deficient; ITT, intent to treat; NR, not reached; PFS, progression-free survival.


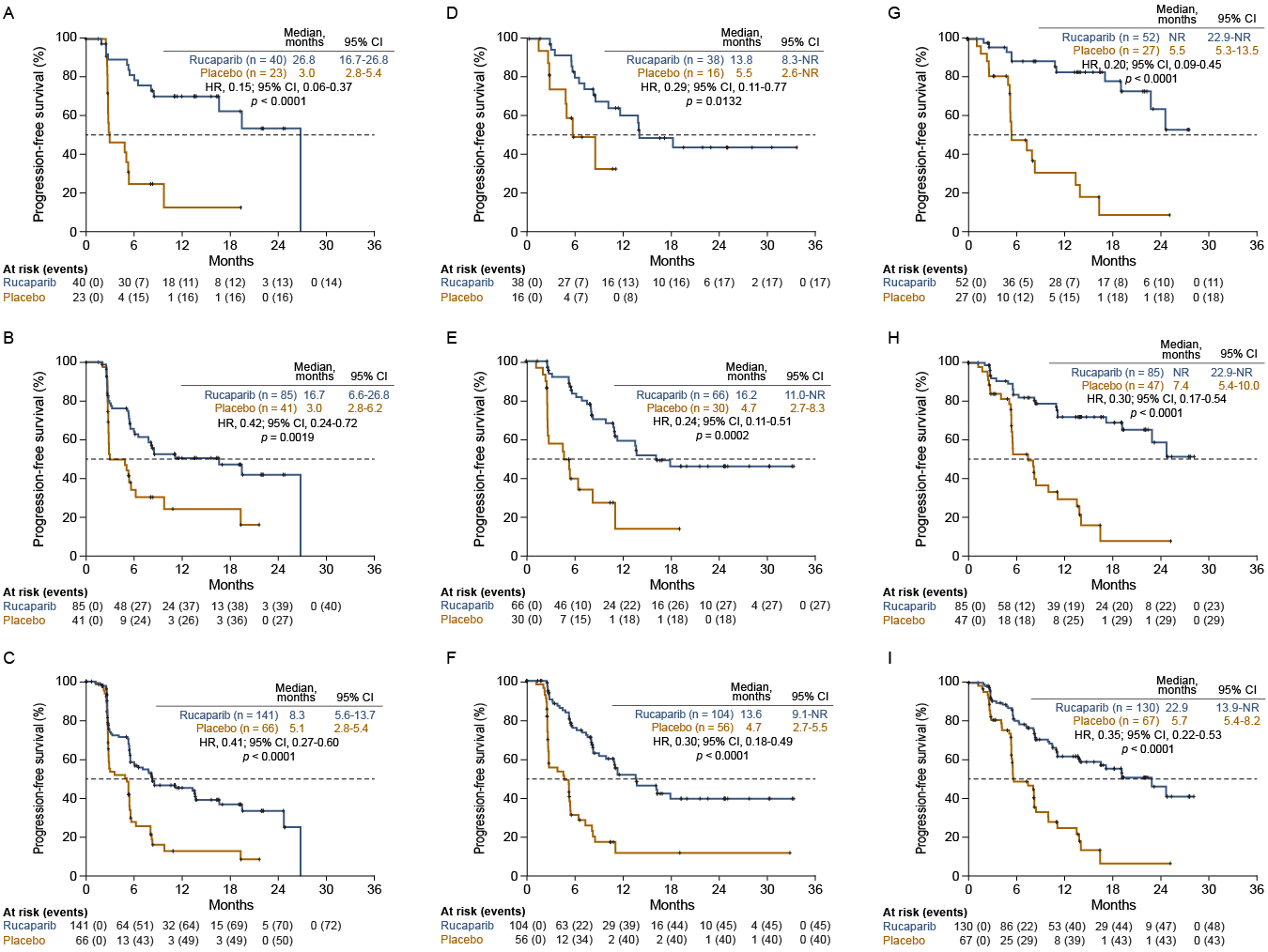

Supplement: Supplementary file 1 — Table S1–S2 [file CAM4-10-7162-s001.docx]
